# Histone H1 Depletion Impairs Embryonic Stem Cell Differentiation

**DOI:** 10.1371/journal.pgen.1002691

**Published:** 2012-05-10

**Authors:** Yunzhe Zhang, Marissa Cooke, Shiraj Panjwani, Kaixiang Cao, Beth Krauth, Po-Yi Ho, Magdalena Medrzycki, Dawit T. Berhe, Chenyi Pan, Todd C. McDevitt, Yuhong Fan

**Affiliations:** 1 School of Biology, Georgia Institute of Technology, Atlanta, Georgia, United States of America; 2 The Petit Institute for Bioengineering and Bioscience, Georgia Institute of Technology, Atlanta, Georgia, United States of America; 3 The Wallace H. Coulter Department of Biomedical Engineering, Georgia Institute of Technology, Atlanta, Georgia, United States of America; The University of North Carolina at Chapel Hill, United States of America

## Abstract

Pluripotent embryonic stem cells (ESCs) are known to possess a relatively open chromatin structure; yet, despite efforts to characterize the chromatin signatures of ESCs, the role of chromatin compaction in stem cell fate and function remains elusive. Linker histone H1 is important for higher-order chromatin folding and is essential for mammalian embryogenesis. To investigate the role of H1 and chromatin compaction in stem cell pluripotency and differentiation, we examine the differentiation of embryonic stem cells that are depleted of multiple H1 subtypes. H1c/H1d/H1e triple null ESCs are more resistant to spontaneous differentiation in adherent monolayer culture upon removal of leukemia inhibitory factor. Similarly, the majority of the triple-H1 null embryoid bodies (EBs) lack morphological structures representing the three germ layers and retain gene expression signatures characteristic of undifferentiated ESCs. Furthermore, upon neural differentiation of EBs, triple-H1 null cell cultures are deficient in neurite outgrowth and lack efficient activation of neural markers. Finally, we discover that triple-H1 null embryos and EBs fail to fully repress the expression of the pluripotency genes in comparison with wild-type controls and that H1 depletion impairs DNA methylation and changes of histone marks at promoter regions necessary for efficiently silencing pluripotency gene *Oct4* during stem cell differentiation and embryogenesis. In summary, we demonstrate that H1 plays a critical role in pluripotent stem cell differentiation, and our results suggest that H1 and chromatin compaction may mediate pluripotent stem cell differentiation through epigenetic repression of the pluripotency genes.

## Author Summary

The epigenome and chromatin play critical roles in stem cell fate determination. Linker histone H1 is a major chromatin structural protein that facilitates higher-order chromatin folding. By analyzing the differentiation capacity of embryonic stem cells (ESCs) that lack multiple H1 subtypes, we find, for the first time, that H1 and higher-order chromatin compaction are required for proper differentiation and lineage commitment of pluripotent stem cells. Triple-H1 null murine ESCs are impaired in both spontaneous differentiation and embryoid body differentiation. Furthermore, triple-H1 null ESCs are compromised in neural differentiation. Finally, we demonstrate that H1 depletion leads to failure of efficient repression of pluripotency gene expression both in embryos and in ESC differentiation. We present evidence that H1 participates in mediating changes of histone marks and DNA methylation necessary for silencing pluripotency gene *Oct4* during stem cell differentiation and embryogenesis. This finding is important because it provides a mechanistic link by which H1 and chromatin compaction may participate in pluripotent stem cell differentiation through repression of pluripotency gene expression.

## Introduction

Pluripotent embryonic stem cells (ESCs) can self-renew and differentiate into diverse cell types, including lineages from all three germ layers present in the adult organism, offering great promise in regenerative medicine in addition to serving as a useful system for developmental biology studies. The epigenome and transcriptional circuitry of pluripotent stem cells have been extensively investigated, and chromatin and epigenetic signatures have emerged as key components in defining and regulating stem cell pluripotency [1]–[4]. Recent reports have associated ESCs with a particularly open, hyperdynamic chromatin and hyperactive global transcription [2], [5], [6], and open chromatin has been suggested as a marker for pluripotency [7], [8]. However, it remains undetermined whether higher order chromatin compaction is required for pluripotent stem cell differentiation and how an open chromatin state impacts stem cell function.

In eukaryotic cells, histones are the major structural proteins that associate with DNA to form chromatin. The basic repeating unit of chromatin is the nucleosome core particle, which consists of an octamer of four core histones (H2A, H2B, H3 and H4) wrapped by 146 bp of DNA [9]. Further compaction of chromatin into higher order structures, such as a 30 nm fiber, is facilitated by binding of H1 linker histones to DNA entry/exit points of nucleosomes and linker DNA between nucleosomes. Reducing the total amount of H1 *in vivo* leads to a relaxed chromatin structure [10]–[12].

The H1 histone family is the most divergent and heterogenous group of histones among the highly conserved family of histone proteins. In mammals, 11 non-allelic H1 subtypes have been identified, including five somatic H1 subtypes (H1a–e), the replacement subtype H1^0^, four germ cell specific H1 subtypes (oocyte specific H1oo, and testis-specific H1t, H1t2, H1LS1) as well as a more recently identified and distantly related subtype H1x [13]. Although the individual depletion of each of the three major somatic H1 subtypes, H1c, H1d and H1e, in mice does not lead to any detectable changes in total H1 levels or obvious phenotypes [14], deletion of H1c, H1d and H1e altogether leads to nearly a 50% reduction of total H1 levels and embryonic lethality with a broad phenotype [15], demonstrating that critical levels of total H1 histones are essential for mouse embryogenesis.

We have previously derived wild-type (WT) and H1c/H1d/H1e triple knockout (H1 TKO) embryonic stem cells from the outgrowth of the inner cell masses of blastocysts attained from intercrosses of H1 heterozygous mutants [10]. We have measured that wild-type ESCs have an H1/nucleosome ratio of 0.46 [10], a much lower level compared with a ratio of 0.75∼0.83 from various differentiated cell types in mouse tissues [11], [15], suggesting that ESCs have a more open chromatin structure compared with differentiated cell types in adult tissues. H1 TKO ESCs have an even lower H1/nucleosome ratio that is close to 0.25, equivalent to 1 H1 per 4 nucleosomes. The compound H1 null ES cells display chromatin decondensation in bulk chromatin [10] and an increased nuclear size [16], offering an ideal system to test the necessity of chromatin compaction on ESC pluripotency and differentiation.

In the current study, we demonstrate, for the first time, that the differentiation capacity of ESCs that lack multiple H1 subtypes is severely impaired. We find that compound H1 null ESCs are more resistant to spontaneous differentiation, impaired in embryoid body differentiation, and largely blocked in neural differentiation. Finally, we present evidence that H1 contributes to efficient repression of the expression of pluripotency factors and participates in establishment and maintenance of epigenetic marks necessary for silencing pluripotency genes during stem cell differentiation and embryogenesis.

## Results

### Loss of H1c/H1d/H1e inhibits spontaneous ESC differentiation

**Figure 1:**
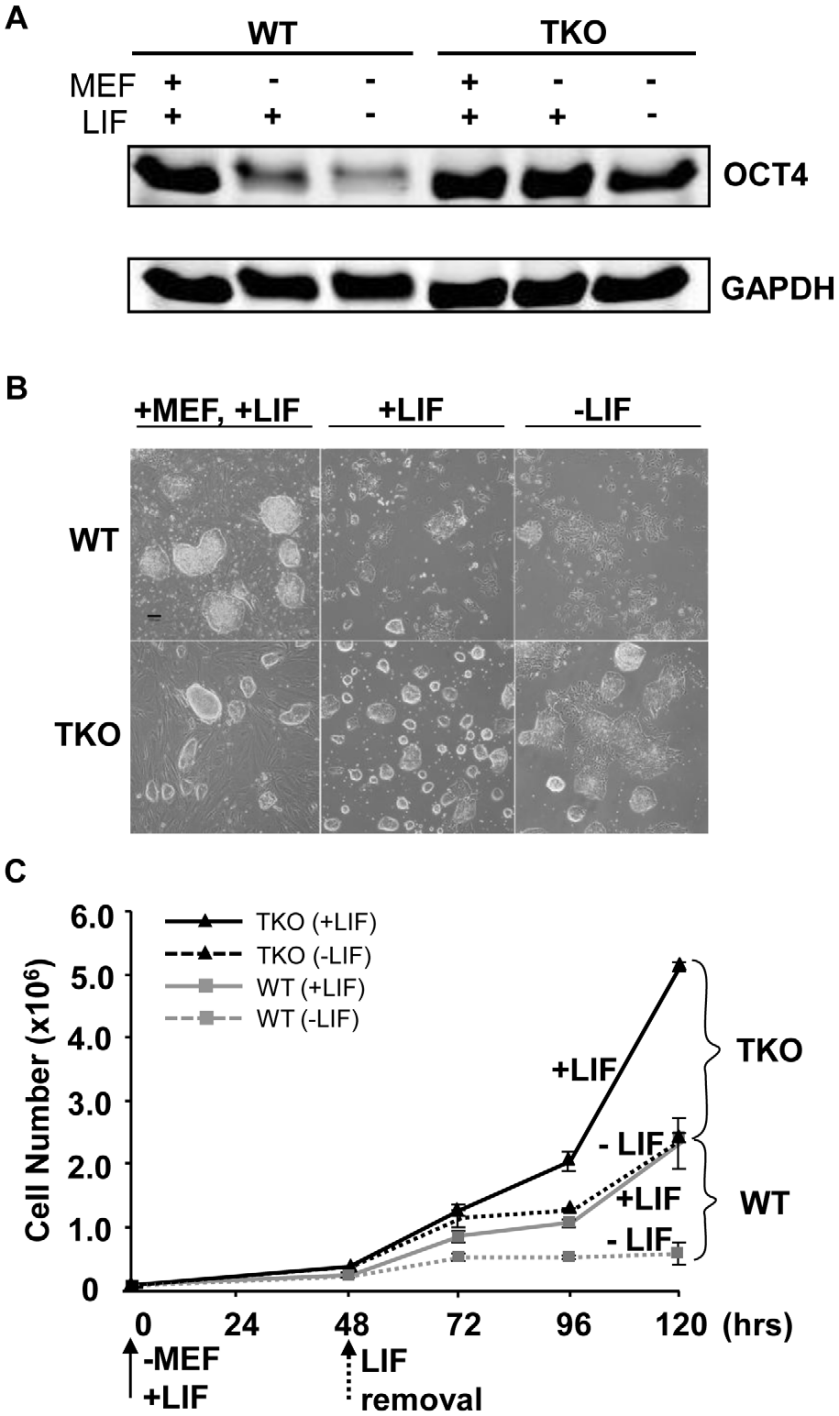
Loss of H1c/H1d/H1e inhibits spontaneous ESC differentiation. (A) Western blot analysis of OCT4 level in WT and H1 TKO ESCs cultured under indicated conditions for 2 days. (B) Phase images of WT and H1 TKO ESCs cultured either on MEF with LIF (left panel), gelatin coated plate with LIF (middle panel), or gelatin coated plate without LIF (right panel) for 2 days. Scale bar: 100 µm. (C) Growth curves of WT and H1 TKO ESCs cultured on gelatin coated plate with or without LIF. Data are presented as average ± S.D.

ESCs exhibit a relatively “open” chromatin structure compared with differentiated cells or lineage committed cells [8]. H1c/H1d/H1e triple null ESCs we derived previously have a significant reduction in total H1 protein levels which leads to further decreased chromatin compaction [10], thus we postulated that loss of H1c, H1d, and H1e may interfere with ESC differentiation. We first compared the spontaneous differentiation tendency of two H1 TKO ESC lines with wild-type littermate ESC lines. Consistent with previous observations [10], H1 TKO ESCs cultured on mitotically inactivated mouse embryonic fibroblast (MEF) feeder cells with media containing leukemia inhibitory factor (LIF) have comparable growth rate to that of wild-type ESCs (data not shown) and normal karyotypes (Figure S1). In addition, H1 TKO ESCs expressed comparable levels of pluripotency factor OCT4 (POU5F1) (Figure 1A), and displayed a similar ESC colony morphology to that of WT ESCs under culture conditions which promote ESC self-renewal (Figure 1B, left panel). However, when cultured in a feeder-free manner on gelatin-coated plates without MEFs, the H1 TKO cells displayed higher levels of OCT4, a more homogeneous, undifferentiated colony morphology, and a higher growth rate than WT ESCs under the same condition (Figure 1A, 1B middle panel, and 1C). Furthermore, upon removal of LIF, the majority of H1 TKO ESCs continued to retain high expression levels of OCT4 (Figure 1A) as well as a tightly packed colony morphology typical of undifferentiated ESCs (Figure 1B, right panel) for a week. In contrast, wild-type ESCs differentiated readily, with approximate 90% of the cells appearing to differentiate by 2 days after LIF removal in feeder free culture, as judged by diminishing OCT4 expression and the loss of a compact colony morphology (Figure 1A, 1B right panel). Removal of LIF reduced the growth of both WT and H1 TKO ESCs (Figure 1C), consistent with LIF's known role in promoting self-renewal and proliferation of ESCs [17]. Collectively, these results suggest that ESCs lacking H1c, H1d, and H1e are more refractory to spontaneous ESC differentiation *in vitro*.

### Loss of H1c, H1d, and H1e impairs EB differentiation

**Figure 2:**
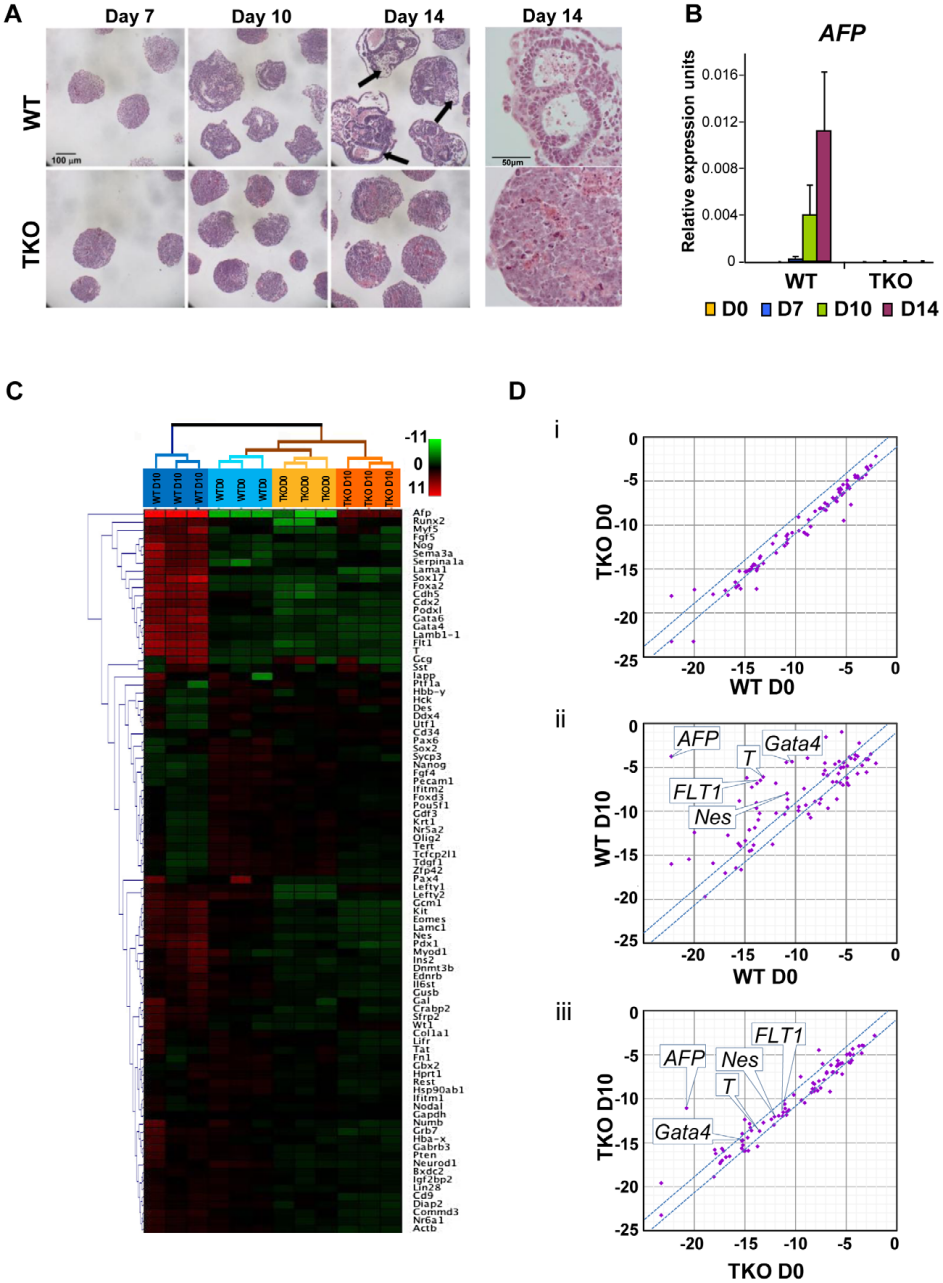
H1c/H1d/H1e triple knockout ESCs are impaired in EB differentiation. (A) Hematoxylin and eosin (H&E) staining of sections of WT EBs (top panels) and H1 TKO EBs (bottom panels) at 7 days, 10 days and 14 days in rotary suspension culture. High magnification images of H&E staining of sections of WT EB (top right) and H1 TKO EBs (bottom right) show that TKO EBs failed to cavitate. WT EBs showed more differentiated morphologies with cysts forming (black arrows). (B) Quantitative RT-PCR analysis of mRNA expression levels of *AFP* in ESCs (day 0) and EBs throughout 14 days of rotary suspension culture. Data were normalized over the expression level of *GAPDH* and are presented as average ± S.D. (C) Hierarchical clustering analysis of qRT-PCR SuperArray gene expression profiling of ESCs (day 0) and EBs (day 10) formed from WT and H1 TKO ESCs. Red, green or black represent higher, lower, or no change in relative expression. (D) Scatter Plot analysis of gene expression comparisons of: (i) WT *vs.* H1 TKO ESCs (day 0); (ii) WT EBs (day 10) *vs.* WT ESCs (day 0); (iii) H1 TKO EBs (day 10) *vs.* H1 TKO ESCs (day 0). X- and y- axes are delta CTs using *GAPDH* to normalize. Genes with more than 2-fold differences lie outside of the blue lines.

To assess whether loss of H1c, H1d and H1e impairs cellular differentiation of any of the three germ layers, we examined the ability of H1 TKO ESCs to form embryoid bodies (EB) using a rotary orbital suspension culture system to induce differentiation *in vitro*. We have previously shown that the rotary suspension culture method offers improved efficiency and homogeneity of embryoid body production compared with the common practice of forming EB aggregates in static suspension culture [18]. During EB culture in serum-containing media, ESCs form aggregates and differentiate into cell types of all three primitive germ layers: endoderm, mesoderm and ectoderm, offering a temporal window to investigate specific defects in lineage differentiation. After 10 days of culture in rotary suspension, the wild-type EBs had a distinct outer endoderm-layer surrounding differentiated cell morphologies representing the three germ layers, including different epithelial cell types and mesenchymal cell populations (Figure 2A). In contrast, although H1 TKO ESCs were able to form putative EBs, most H1 TKO EBs appeared blocked in the differentiation process in rotary suspension culture, forming undifferentiated masses of stem cells that lacked cavity formation and other types of differentiated structures even after prolonged culture in rotary suspension (up to 14 days) (Figure 2A). Quantitative RT-PCR analyses also indicated that the expression of differentiation markers, such as the endoderm marker, alpha-fetoprotein (*AFP*), was drastically increased in WT EBs, but significantly curbed in H1 TKO EBs (Figure 2B). The mRNA levels of other lineage specific markers, including mesoderm markers, such as the cardiac transcription factor *Nkx2.5*, and the sarcomeric muscle marker, alpha myosin heavy chain (*αMHC*), also progressively increased over time in WT EBs, but were not detected at similar levels in H1 TKO EBs (Figure S2A).

To gain a more comprehensive view of the scope of genes affected by linker histone H1 depletion during differentiation, we performed quantitative PCR SuperArray analysis of wild-type and H1 TKO cells at the start (day 0) and the end point (day 10) of rotary suspension culture. The genes analyzed included pluripotency genes as well as important developmental genes for transcription factors and signaling molecules for all three germ layers. WT and TKO cultures at day 0 displayed very few differences in gene expression and their gene expression profiles clustered most similarly in hierarchical cluster analysis (Figure 2C, 2Di, and Figure S2Bi). WT EBs differentiated as expected with significant increases of many differentiation markers and decreased expression of pluripotency associated genes (Figure 2C, 2Dii, and Figure S2Bii). In contrast, H1 TKO EBs exhibited very similar gene expression signatures to those of ESCs and had less expression changes during differentiation compared with that of WT EBs. (Figure 2C, 2Diii, and Figure S2Biii, S2C), suggesting that the lack of H1c, H1d and H1e leads to diminished changes of transcriptional reprogramming during differentiation. The levels of ectoderm markers, such as *Nestin* (*Nes*), mesoderm markers, such as *Brachyury* (*T*) and *FLT1*, and endoderm markers, such as *AFP* and *Gata4*, were all markedly less or failed to be expressed in H1 TKO EBs (Figure 2C and Figure S2C), indicating that differentiation to all three germ layers was suppressed.

### H1 is required for neural differentiation of embryonic stem cells

**Figure 3:**
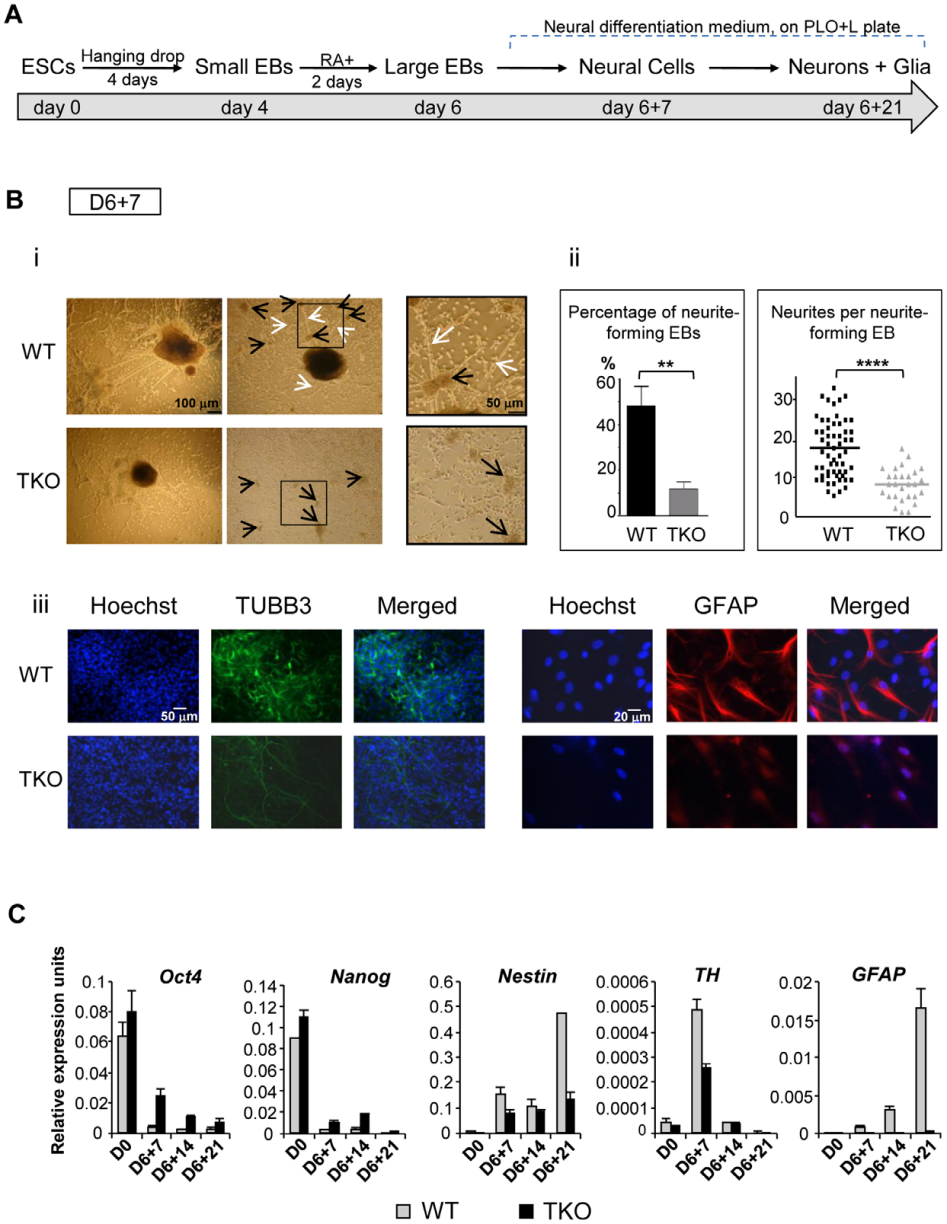
H1 TKO ESCs fail to undergo neural differentiation. (A) Neural differentiation scheme for ESCs. (B) Characterization of WT and H1 TKO cultures on day 6+7 under neural differentiation protocol. i). Phase contrast images shows that H1 TKO mutants were unable to adequately form neurites and neural networks. Right panels: zoom-in images of the areas encircled with black rectangles. Scale bar: 100 µm (left panels) and 50 µm (right panels). ii). Left panel: Percentage of neurite-forming EBs. Numbers were averaged from 6 experiments. 80 EBs were counted per experiment. Right panel: Numbers of neurites per neurite-forming EB. Number of neurites was counted from EBs that produced neurites. 58 and 28 neurite-forming EBs from respective WT and TKO were selected and counted for neurite numbers. **: P<0.01; ****: P<0.0001. iii). Immunostaining for expression of TUBB3 and GFAP. Nuclei were stained with Hoechst 33342. Scale bars: 50 µm (left panels) and 20 µm (right panels). Results are representative of three independent experiments. (C) H1 TKO ESCs were unable to adequately repress the pluripotency genes and to efficiently induce the expression of neural genes. Expression levels of pluripotency genes (*Oct4* and *Nanog*), neural marker (*Nestin*), neuronal marker (*Tyrosine hydroxylase (TH)*), astrocyte marker (*GFAP*) from WT and H1 TKO cultures at indicated days in differentiation cultures were determined by qRT-PCR. Data were normalized over the expression level of *GAPDH* and are presented as average ± S.D.

To further investigate if and when H1 impacts cell differentiation in a specific lineage, we induced differentiation of H1 TKO ESCs under a neural differentiation regimen established using *all-trans* retinoic acid (RA), which is known to induce neural differentiation in ESCs [19], [20]. EBs were prepared using the hanging-drop method, and day 4 EBs were collected and treated with RA for additional two days followed by further differentiation with neural differentiation media on poly-L-ornithine and laminin (PLO+L) coated tissue culture plates (Figure 3A). By day 6+7 of this *in vitro* neural differentiation scheme, neural cells were clearly established and neurite outgrowth from EBs was seen with neuronal cell proliferation. Neurites are enriched in cylindrical bundles of microtubules, made primarily of β-III tubulin (TUBB3) protein, extending from the body of all neurons, finally differentiating into an axon or a dendrite [21]. However, at this time point, neural differentiation of WT and TKO ES cells exhibited several striking differences.

While neurite-formation was efficient in WT culture with bundles of neurites cylindrically extending from EB to adjacent EB, H1 TKO EBs had much less neurite outgrowth (Figure 3Bi, 3Bii). Approximately 50% of WT EBs plated for neural differentiation formed neurites compared to only about 10% of H1 TKO EBs forming neurites (Figure 3Bii, left panel). Furthermore, those 10% TKO EBs that were capable of forming neurites only produced on average 8 neurites per EB, whereas each WT EB had on average 18 neurites (Figure 3Bii, right panel). During *in vitro* neural differentiation, neurons aggregated into mounds of cells forming neuronal clusters (Figure 3Bi; black arrows), connected by bundles of neurites (Figure 3Bi; white arrows), forming a network pattern. While WT cultures showed formation of a neural network with neural clusters inter-connected by bundles of neurites, H1 TKO cultures failed to develop such an extensive intercellular network (Figure 3Bi, ii), evidenced by smaller neuronal clusters with negligible inter-connecting neurites. This was further confirmed with immunofluorescence detection of TUBB3 protein expression, and minimal TUBB3 staining was seen in H1 TKO cultures (Figure 3Biii). It appeared that both neurite formation and outgrowth were limited in H1 knock-out mutants, affecting the ability of neurons to form neural networks. We also noted that TKO cultures yielded markedly less glial cells as revealed by much fewer GFAP positive astrocytes in comparison with WT cultures (Figure 3Biii). Since glial cells are essential for the normal growth and development of neurons, the near-lack of glial cells in TKO cultures may contribute to the poor development of TUBB3 positive neuronal cells from TKO EBs.

To examine whether the aforementioned defects of the H1 TKO cultures represent a temporary delay or a blockage in neural differentiation, we cultured the cells for an additional 14 days under neural differentiation conditions. As expected, the neural marker (*Nestin*) and the astrocyte marker (*GFAP*) were efficiently and progressively induced in WT cell cultures, and the neuronal gene *Tyrosine hydroxylase (TH)* peaked at day 6+7 when neuronal proliferation occurred (Figure 3C). In contrast, the expression levels of neural genes were significantly curtailed in H1 TKO cultures, suggesting the lack of progression in neural differentiation of H1 TKO culture (Figure 3C). Furthermore, we observed that pluripotency genes *Oct4* and *Nanog* were expressed at higher levels in TKO than WT throughout the differentiation process (Figure 3C). These data suggest that H1 TKO cells are largely blocked in neural differentiation.

### Levels of H1 increase progressively during differentiation

**Figure 4:**
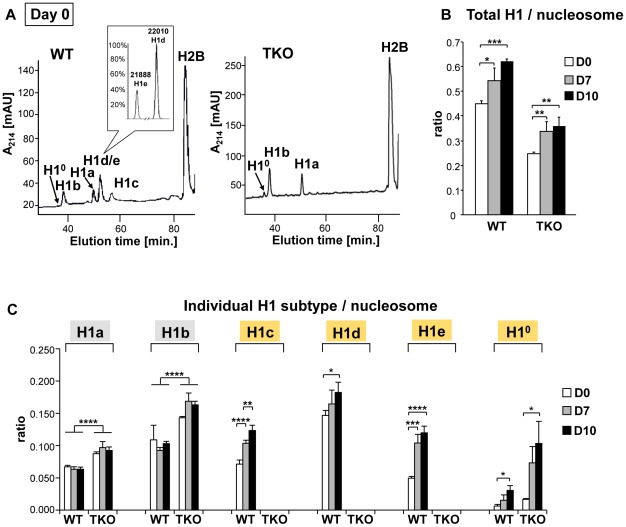
Expression profiles of linker histones in WT and H1 TKO cultures during EB differentiation. (A) Reverse-phase HPLC and Mass Spectrometry (inset) analysis of histones from WT and H1 TKO ESCs. X axis: elution time; Y axis: absorbency at A_214_. mAU, milli-absorbency units. Inset shows the relative signal intensity of H1d and H1e mass spectral peaks in the H1d/H1e fraction collected from HPLC eluates of WT histones. (B,C) H1/nucleosome ratio of the total H1 (B) and individual H1 subtype (C) during EB formation and differentiation. Day 0, day 7 and day 10 of EB cultures were collected and HPLC analyses as shown in (A) were performed. The ratio of total H1 (or individual H1 subtype) to nucleosome was calculated as described in Materials and Methods. Values are means ± S.D., n = 4. *: P<0.05; **: P<0.01; ***: P<0.001; ****: P<0.0001.

To address the mechanisms by which H1 modulates differentiation, we first examined the expression profile of linker histone H1 subtypes during EB formation and differentiation of wild-type ESCs. Histones from wild-type, H1 TKO ESCs and EBs were isolated at various time points during differentiation, and the levels of individual H1 subtype proteins as well as the H1 to nucleosome ratio were quantified from HPLC and mass spectrometry analysis as described previously [15], [22], [23]. In ESCs (day 0), H1^0^ was nearly undetectable in WT cells but was increased in H1 TKO cultures as we observed previously (Figure 4A and [10]). Upon EB differentiation, the levels of H1c, H1d and H1e and H1^0^ in WT cultures were all progressively increased over time, with the total H1 to nucleosome ratio elevated nearly 40% from 0.45 for ESCs to 0.62 for day 10 EBs (Figure 4B, 4C). Consistent with HPLC analysis, Western blotting showed that levels of total H1 and H1^0^ were increased (Figure S3). The cumulative increase in the protein levels of H1c, H1d and H1e was responsible for 87% of the increase in the total H1 levels during differention (data not shown). Despite less abundant than H1d, H1c and H1e were significantly increased (P<0.001), and H1e levels in differentiated EBs were over 2-fold of that in undifferentiated ESCs (Figure 4C). The protein levels of H1a and H1b remained constant during differentiation, indicating that H1a and H1b were not responsible for the increase of total H1 during ESC differentiation. Albeit higher than that in TKO ESCs (0.25), the ratio of total H1 to nucleosome in day 10 TKO EBs (0.36) remained lower than the ratio in WT ESCs (0.45) (Figure 4B). The increase in the total H1 level in TKO EBs compared with ESCs was largely due to the increase in the level of H1^0^ (Figure 4B, 4C, and Figure S3), indicating H1^0^ being the major H1 subtype upregulated in the face of deficiency of H1c, H1d, and H1e, in both ESCs and EBs. These results show that the levels of H1c, H1d, H1e and H1^0^ are elevated significantly during embryonic stem cell differentiation, and that the H1 TKO EB has a total H1 level lower than the WT ESC.

### H1c/H1d/H1e is necessary for efficient transcriptional repression of pluripotency genes *Oct4* and *Nanog* during embryogenesis and ESC differentiation

**Figure 5:**
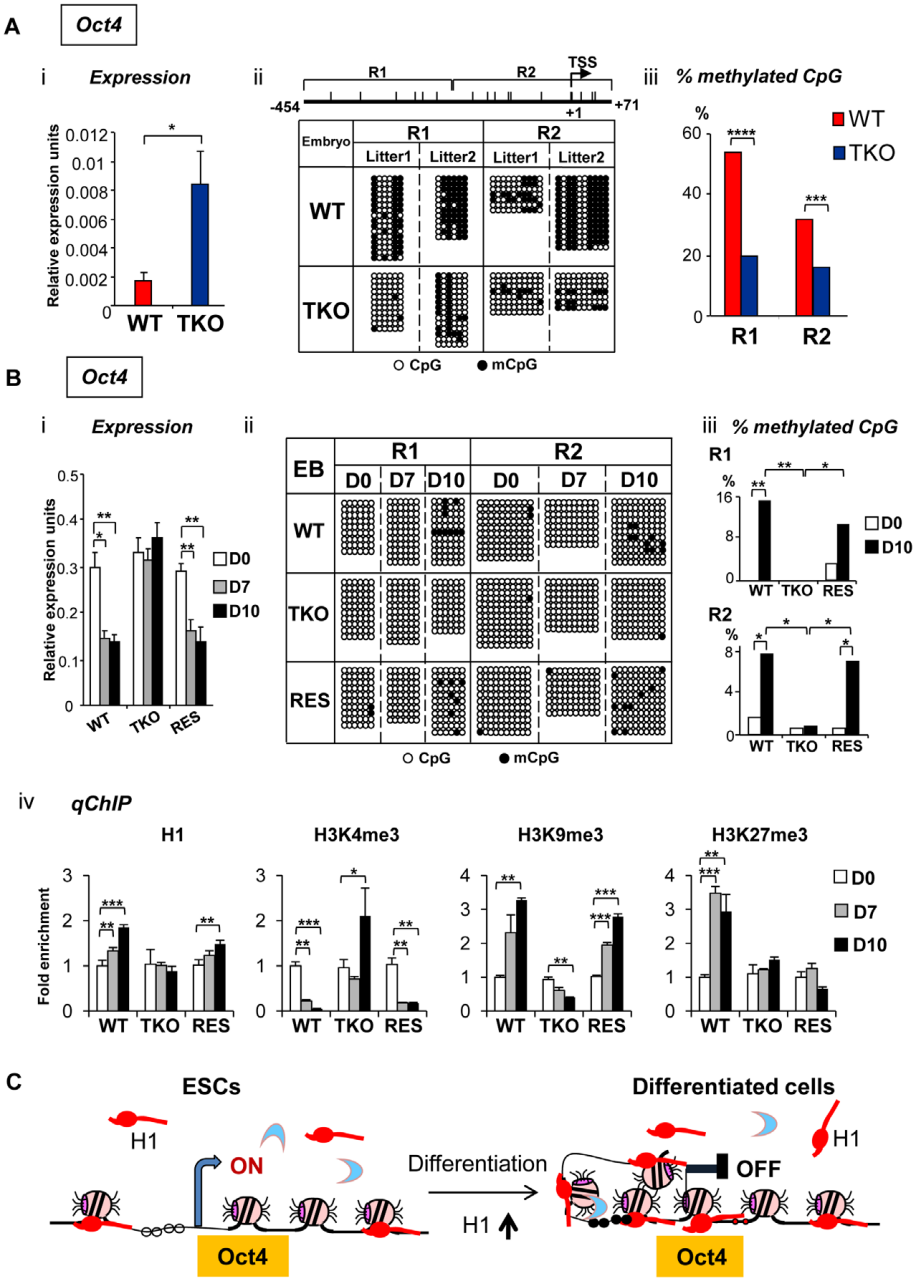
H1 is necessary for stable repression of *Oct4* pluripotency gene during embryogenesis and ESC differentiation. (A) Elevated *Oct4* expression and hypomethylation of CpG sites at *Oct4* promoters in H1 TKO embryos compared with littermates at E8.5. (i) qRT-PCR analysis of mRNA expression levels of *Oct4*. Values are means ± SEM, n = 5 for each genotype. Expression levels were normalized over *GAPDH*. *: P<0.05. (ii) Bisulfite sequencing analysis of DNA methylation status at *Oct4* promoter regions. Results of two wild-type and two knockout E8.5 embryos are shown. The positions of CpG sites analyzed are depicted schematically as vertical ticks on the line. TSS: transcription start site. (iii) Percentage of methylated CpG sites at *Oct4* promoter regions in WT and H1 TKO embryos. Statistical analysis was performed using Fisher's exact test. ***: P<0.001; ****: P<0.0001. (B) Analysis of expression and epigenetic marks at *Oct4* pluripotency gene during EB differentiation in rotary suspension culture. Analyses of expression (i), DNA methylation (ii), % of mCpG (iii); and occupancy of H1 and three histone marks (iv) of *Oct4* in WT, H1 TKO and RES cells during EB differentiation. Relative expression levels were normalized over *GAPDH*. Relative fold enrichment is calculated by normalizing the qChIP values (as described in Material and Methods) of ESCs (day 0) or EBs at each time point by that of WT ESCs (WT D0). Values are presented as mean ± S.D. *: P<0.05; **: P<0.01; ***: P<0.001. (C) Model for H1 in repression of *Oct4* during ESC differentiation. ESCs have low H1 content with an relatively “open” chromatin. During differentiation, total H1 content increases, which facilitates local chromatin compaction at *Oct4* gene and contributes to establishment and/or maintenance of epigenetic changes necessary for stable silencing of *Oct4* pluripotency gene.

The results from the aforementioned experiments suggest that H1c/H1d/H1e triple null ESCs are less effective than WT ESCs in repressing the expression of pluripotency genes, such as *Oct4* and *Nanog*, during spontaneous differentiation, rotary suspension differentiation, and neural differentiation *in vitro* (Figure 1A, Figure 2C, and Figure 3C). Therefore, we next investigated if H1 contributes to stable repression of pluripotency gene expression *in vivo* during embryogenesis. *Oct4* is expressed in undifferentiated cells in the preimplantation embryo, and is progressively down-regulated in differentiating embryonal cells during gastrulation, becoming restricted to germ cell precursors after E8.5 [24], whereas *Nanog* expression is largely downregulated after E4.5 [25]. We analyzed expression of *Oct4* and *Nanog* from E8.5 embryos, when many of the surviving TKO embryos appeared comparable to WT littermates. E8.5 embryos were harvested from intercrosses of H1c/H1d/H1e triple heterozygotes and the expression levels of *Oct4* and *Nanog* in TKO and WT embryos were analyzed from three litters using quantitative RT-PCR. On average, expression levels of *Oct4* and *Nanog* in TKO embryos were more than 4-fold of that from WT littermate controls (Figure 5Ai, Figure S4A), indicating that depletion of H1 impairs repression of the expression of pluripotency factors in E8.5 embryos *in vivo*.

DNA methylation of cytosine nucleotide at CpG sites within gene promoter regions contributes to stable gene silencing, and thus is a key determinant in regulating the expression of pluripotency genes [26], so we asked if the DNA methylation status at the *Oct4* and *Nanog* promoters is affected in H1 TKO embryos. Results from bisulfite sequencing analysis demonstrated that the extent of CpG methylation at the *Oct4* promoter region was markedly reduced in triple-H1 null embryos in comparison with corresponding wild-type littermates (Figure 5Aii), whereas the level of DNA methylation (percent methylation of analyzed CpGs) at *Nanog* promoter did not display differences between WT and H1 TKO embryos (Figure S4B, S4C). This suggests that H1 participates in establishing and/or maintaining CpG methylation at *Oct4* promoter during embryogenesis.

To further investigate the mechanisms by which H1 regulates pluripotency genes during ESC differentiation, we analyzed the epigenetic profiles of the *Oct4* and *Nanog* genes during EB differentiation in rotary suspension culture. We demonstrated previously that this method produces a large quantity of homogeneous EBs that progressively differentiate [18], thus the sequential epigenetic events can be readily followed. Expression of *Oct4* and *Nanog* was reduced during continuous suspension culture for WT cultures, but remained high in TKO EB cultures (Figure 5Bi, Figure S6A). DNA methylation analysis by bisulfite sequencing indicated that WT EBs had an increase in the sporadic DNA methylation at specific CpG sites throughout the *Oct4* proximal promoter region at day 10 (P = 0.002 and 0.036 for the respective R1 and R2 regions) (Figure 5Bii, iii), whereas TKO EBs remained completely unmethylated at these sites. On the other hand, *Nanog* promoter region remained unmethylated throughout the differentiation in both WT and H1 TKO cultures (Figure S6B).

To further investigate the effect of H1 levels in affecting expression and DNA methylation of pluripotency genes in EB differentiation, we generated “rescue” cell lines (referred to as “RES”) by stably overexpressing exogenous H1d in the H1 TKO cells (Figure S5A). RES cells had a H1/nucleosome ratio of 0.31 (Figure S5B), displayed a normal karyotype (Figure S5C), and were able to differentiate into EBs with cystic structures which were observed in WT, but not in TKO, EBs (Figure S5D). RES EBs had elevated expression of differentiation markers, such as *AFP* and *Nkx2.5* (Figure S5E) and reduced expression of *Oct4* and *Nanog* pluripotency genes upon differentiation (Figure 5Bi and Figure S6A), suggesting that the expression of exogenous H1d alleviates the differentiation defects and restores the repression of pluripotency factors in H1 TKO EBs. In addition, the percent of methylated CpG was increased in RES EBs to a level comparable to that of WT EBs at the same time points, suggesting that reintroduction of H1d into the H1 TKO ESCs is able to reestablish DNA methylation and the stable repression of the *Oct4* gene in differentiating EBs (Figure 5Bii, iii).

We next analyzed the status of H1, H3K4me3, H3K9me3 and H3K27me3 at the promoters of pluripotency genes *Oct4* and *Nanog* by quantitative chromatin immunoprecipitation (qChIP). Whereas H1 occupancy at *Oct4* promoter increased in WT and RES cultures during differentiation, it remained unchanged in H1 TKO EBs (Figure 5Biv). It is interesting to note that the occupancy of the replacement subtype, H1^0^, at *Oct4* promoter was markedly increased in both WT and RES cultures but only mildly elevated in H1 TKO cultures (Figure S7), suggesting that efficient binding of H1^0^ at *Oct4* promoter may be facilitated by sufficient amount of other somatic H1s. Furthermore, wild-type and RES EBs displayed decreasing levels of the active histone mark H3K4me3 accompanied with a significant increase in the levels of the repressive histone mark H3K9me3, at promoter regions of pluripotency genes *Oct4* and *Nanog* upon EB differentiation (Figure 5Biv and Figure S6C). In contrast, H1 TKO EBs did not display similar or significant changes in the levels of these histone marks at the same promoter regions (Figure 5Biv and Figure S6C). Levels of H3K27me3, another repressive histone mark, were significantly increased in WT cultures during differentiation at *Oct4* promoter, while such increases were not detected at H1 TKO or RES EBs (Figure 5Biv).

These analyses suggest that the increase of H1 levels and the changes in histone modifications, such as H3K4me3, H3K9me3 and H3K27me3, precede DNA methylation establishment in mediating *Oct4* gene silencing during EB differentiation. Overall, the results indicate that lack of H1c, H1d and H1e impairs the establishment or maintenance of epigenetic changes in DNA methylation and histone modifications that are necessary for stable repression of pluripotent transcription factor *Oct4* in differentiated cells (Figure 5C).

## Discussion

Embryonic stem cells, derived from the inner cell mass of the blastocyst stage mammalian embryos [27], [28], can self-renew nearly indefinitely in culture and give rise to all cell types of the three germ layers, ectoderm, mesoderm and endoderm, during differentiation. ESCs possess distinctive transcriptional regulatory circuits and chromatin signatures that are critical for maintaining pluripotency and self-renewal [29], [30]. Recent studies suggest that ESCs exhibit a relatively “open” chromatin state, and during differentiation, heterochromatin formation increases [2], [8], [31]. However, whether this “open” chromatin state is necessary for pluripotency and whether the compaction of chromatin is required for ESC differentiation remain to be addressed.

Linker histone H1 is the major chromatin architectural protein in mediating higher order chromatin folding. H1 TKO ESCs have an H1/nucleosome ratio of 0.25, equivalent to 1 H1 per 4 nucleosomes, a nearly 50% reduction in total H1 levels in comparison with WT ESCs [10]. The H1 level is especially low in H1 TKO ESCs when compared with an H1/nucleosome ratio of 0.75∼0.8 in differentiated cell types from various adult tissues [11], [15]. H1 TKO ESCs have globally decondensed chromatin [10], offering an approachable means to examine the effect of chromatin decondensation on ESC pluripotency and differentiation. H1 TKO ESCs maintain ESC colony morphology, express pluripotency factors (Figure 1A), propagate and self-renew normally as wild-type ESCs, suggesting that a more “open” chromatin structure than normal WT ESCs does not interfere with the “basal” state of ESCs, and may even promote the maintenance of this primitive state. This prediction is consistent with the fact that H1 TKO ESCs are easier to maintain and have sustainable OCT4 pluripotency factor expression and robust growth even under conditions normally promoting spontaneous differentiation, such as culturing ESCs in the absence of LIF and feeder cells for a prolonged period. ESCs are found to have hyperdynamic chromatin with loosely bound major chromatin architectural proteins, such as H1 and HP1 [6]. A more “open” chromatin in H1 TKO ESCs may suggest a more dynamic chromatin structure due to the lack of structural constraints. However, it is not clear at present whether the remaining H1 proteins in H1 TKO ESCs undergo a change in post-translational modifications, such as phosphorylation, which would change the binding affinity of these remaining H1 subtypes to chromatin [32], [33]. We also note the considerable amount of H1s remaining in these TKO ESCs, thus further reducing H1 amount by knockout or siRNA could help determine if a minimal level of H1 is required to permit self-renewal of ESCs.

While a significant reduction in H1 levels does not interfere with ESC self-renewal, it appears to clearly impair ESC differentiation. This is manifested in static culture conditions that promote spontaneous ESC differentiation, in a rotary suspension culture system which induces highly reproducible and robust EB formation and differentiation [18], [34], as well as in a well defined neural differentiation regimen. H1 TKO EBs formed in rotary culture have a reduced level of activation of many developmental genes and markers from all three germ layers, suggesting that the effects of H1 depletion on differentiation and cell fate decision broadly impact early developmental gene expression. This may explain why only 50% of H1 TKO embryos are present at E7.5 [15]. Furthermore, H1 TKO ESCs are defective in forming neuronal cells, glial cells, and lack formation of neural network, which are essential for nervous system development *in vivo*. Total levels of H1 increases progressively in EB formation and differentiation, suggesting an increasingly more condensed chromatin state during EB differentiation in WT cultures. H1 TKO EBs have an H1 to nucleosome ratio lower than WT ESCs. The fact that H1 TKO ESCs cells are unable to execute normal differentiation programs suggests that an especially low H1 level (and the resulting more open chromatin structure [10]) impairs ESC pluripotency and differentiation. Thus, elevated levels of the total H1 amount as well as a more compact chromatin are not mere consequences of differentiation processes, but a necessity to enable it to proceed normally.

H1c, H1d, H1e and H1^0^ are four H1 subtypes that increase significantly during ESC differentiation. H1x, although whose mRNA expression has been reported to increase during differentiation of human ESCs and embryocarcinoma cells [35], [36], is not detected in HPLC profiles of both WT and TKO ESCs throughout differentiation despite a 2-fold increase in mRNA levels in TKO ESCs compared with WT ([10] and data not shown). Thus, this more distantly related H1 subtype (H1x) is present at a negligible level compared with the 6 somatic H1 subtypes (H1a-e and H1^0^) in ESCs and EBs. In contrast, H1a and H1b are abundantly present in ESCs, together accounting for one third of total H1 content in WT ESCs. Although both H1a and H1b increase approximately 50% in TKO ESCs upon depletion of H1c, H1d and H1e, the levels of H1a and H1b do not increase during EB differentiation of WT or TKO cultures. Thus, H1c, H1d, H1e, and H1^0^, but not H1a and H1b, are likely to be the major contributors for the effects of H1 on ESC differentiation and repression of pluripotency genes during ESC differentiation. In particular, H1^0^, a subtype highly expressed in differentiated cells and tissues [13], progressively increases in bulk chromatin and at the *Oct4* promoter during EB differentiation and largely accounts for the increase in total H1 levels in TKO EBs during differentiation (Figure 4, Figure S3, and Figure S7). Thus it would be very interesting to investigate if further deletion of H1^0^ in the face of H1 TKO will result in a complete inhibition of ESC differentiation. Nevertheless, none of these four H1 subtypes alone appears to be required for mouse ESC differentiation, because knockout mice with deletion of one of these four H1 subtypes develop normally [14], [37], suggesting that the differentiation defects we observed here are more likely caused by a marked reduction of total H1 content in H1 TKO cells. Furthermore, we show that a partial rescue of H1 content by reintroduction of H1d into TKO cells mitigates the impairment of differentiation. Together, we surmise that a potential threshold of H1 levels, but not necessarily a specific H1 subtype, is required for proper ESC differentiation.

The effects of H1 depletion on gene expression in EBs are significant and wide-spread, drastically affecting many genes (Figure 2C, 2D, and Figure S2C), in sharp contrast to the limited number of genes with altered expression in H1 TKO ESCs [10]. It is conceivable that H1 depletion in ESCs and a marked decondensation of the chromatin pose little effects on the “basal” state of ESCs, but more so on impairing the capability of ESCs to transit to differentiated cells which exhibit more compact chromatin. Nevertheless, the influence of H1 on many developmental genes in EBs could be a secondary effect resulting from the lack of effecient repression of pluripotency gene expression, such as *Oct4* and *Nanog*, which associate with repressor complexes to silence developmental genes [38]. The effects might also be caused by misregulation of multiple key developmental genes required for normal differentiation to proceed. It is interesting to note that 50% of H1 TKO embryos are able to progress to mid-gestation, suggesting that early differentiation in three germ layers *in vivo* is possible for some TKO embryos [15]. Consistently, H1 TKO ES cells are capable of forming EBs (Figure 2), albeit mostly impaired in differentiation, and teratomas that contain a small fraction of cells differentiated into the three germ layers (data not shown). The impairment of ESC differentiation *in vitro* yet survival of some knockout embryos to mid-gestation stage is reminiscent of several other knockouts of ubiquitously expressed proteins that bind and modify chromatin [8], [39]–[41], which probably reflects more heterogenous cell populations and conditions *in vivo*.

Importantly, we discovered that, compared with WT ESCs, the H1 TKO cells fail to effectively silence the expression of pluripotency genes *Oct4* and *Nanog*, which are critical for pluripotency [42], [43]. We believe that this effect of H1 on repression of *Oct4* is direct because 1) *Oct4* expression is higher in H1 TKO compared with WT both *in vivo* in embryos and *in vitro* using three differentiation schemes for ESCs and EBs, although the degree of effects varies according to different differentiation schemes employed; 2) reconstitution of H1d into H1 TKO ESCs restores the effective repression of expression and dynamic changes in histone modifications and DNA methylation levels during differentiation; 3) the level of H1 is cumulatively increased at the *Oct4* promoter during differentiation of WT, but not of H1 TKO, cultures. We suggest that the H1 occupancy at *Oct4* promoter in ESCs could be the basal/minimal level for detection by qChIP assay, as H1 has been found to be relatively depleted from active promoters compared with other regions [44], [45]. Interestingly, qChIP analysis showed that the association of H1^0^ at *Oct4* promoters was significantly higher in RES cells than TKO cells (Figure S7), suggesting that the presence of sufficient H1 proteins may facilitate H1^0^ binding. We surmise that the progressive increase of H1c, H1d and H1e during differentiation and the increased H1 occupancy at *Oct4* promoter lead to a transition to a more condensed local chromatin structure necessary for stable silencing of *Oct4* during differentiation (Figure 5C). These results together with the observation that OCT4 is present at the promoters of several H1 subtypes in human ESCs [29], [35] suggest a potential feedback loop between OCT4 and H1 in stem cell fate determination.

Interestingly, we found that CpG methylation of *Oct4* promoter in H1 TKO embryos is significantly reduced compared with wild-type littermates. Although less pronounced in EB differentiation, the effects of H1 depletion on DNA methylation at *Oct4* promoter are also apparent in day 10 EBs. This observation reinforces the link between H1 and DNA methylation, which was initially discovered at imprinting control regions (ICRs) of *H19* and *Gtl2* loci [10] and later at regulatory regions of the immunoglobin heavy chain locus and homeobox *Rhox* gene cluster [46], [47]. Future studies on how DNA methylation changes at these regions in H1 TKO ESCs during differentiation will provide additional insights on dynamic profiles of DNA methylation upon differentiation in the face of minimal level of H1 and/or open chromatin structure.

H1 TKO EBs do not exhibit the opposite changes in the levels of the active histone mark (H3K4me3) and the repressive histone mark (H3K9me3) at promoters of *Oct4* and *Nanog* that normally occur in wild-type EBs during differentiation. Interestingly, we did observe significant changes in the levels of histone modifications in wild-type EBs at day 7 in rotary culture, before an increase in DNA methylation levels occurred at *Oct4* promoter. This result reinforces the notion that DNA methylation is a slower mark to establish compared with histone marks [48]. It is noteworthy that the levels of DNA methylation at the *Nanog* promoter do not display a difference in WT and H1 TKO embryos at day 8.5 and are not altered during EB differentiation, suggesting that DNA methylation is unlikely to be responsible for gene expression changes of *Nanog* during this period of time.

Our results suggest a role of H1 and chromatin compaction in epigenetic regulation of the pluripotency gene *Oct4*, likely mediated through DNA methylation and histone modifications. To our knowledge, this represents a novel mechanistic link by which bulk chromatin compaction is directly linked to pluripotency, by participating in repression of the pluripotency genes. In ESCs, DNMT3b has been shown to interact with H1 [49]. *In vitro* studies demonstrated that H1 interacts with HP1 [50], [51] which can in turn bind to SUV39H which methylates H3K9. Moreover, H1 has been shown *in vitro* to stimulate the activity of PRC2 toward methylation of H3K27me3 when H1 is incorporated into nucleosomes [52], and we have also observed interactions between H1 and PRC2 components in ESCs (Cao, Ho, Lasater, and Fan, unpublished observation). Therefore, we envision that during ESC differentiation, H1 levels increase, which may facilitate the recruitment of DNMTs, SUV39H and PRC2 to *Oct4* promoter, promoting the establishment and/or maintenance of repressive epigenetic modifications and silencing the expression of this pluripotency gene (Figure 5C).

In summary, we have demonstrated that loss of linker histone subtypes H1c, H1d, and H1e impairs embryonic stem cell differentiation. Furthermore, our results indicate that H1 contributes to silencing of pluripotency factors and participates in mediating changes in DNA methylation and histone marks necessary for silencing of pluripotency genes during differentiation. Thus, modulating the levels of H1 linker histones and chromatin compaction may potentially serve as a new strategy for regulating stem cell pluripotency.

## Materials and Methods

### Embryonic stem cell culture

ESC lines derived from H1 TKO and wild-type littermates were expanded on mitotically inactivated mouse embryonic fibroblasts feeder layers and cultured feeder-free on tissue culture-treated dishes (Corning) pre-adsorbed with gelatin (Sigma, 0.1% solution in ddH_2_O) prior to embryoid body differentiation studies. ESC culture media consisted of Dulbecco's modified Eagle's medium (DMEM) (Invitrogen) supplemented with 15% fetal bovine serum (FBS) (Hyclone), 100 U/ml penicillin, 100 µg/ml streptomycin and 0.25 µg/ml amphotericin (Mediatech), 2 mM L-glutamine (Mediatech), 1× MEM non-essential amino acids (Mediatech), 0.1 mM β-mercaptoethanol (Fisher Chemical), and 10^3^ U/ml of leukemia inhibitory factor (LIF; ESGRO, Chemicon). Cultures were re-fed with fresh media every other day, and passaged every 2–3 days prior to reaching 70–80% confluence. For spontaneous differentiation studies, 2×10^5^ cells were seeded in each well of 6-well plate at day 0 on gelatin coated plate without feeder layer, cultured with media without LIF, and harvested at indicated time points. Cell numbers were determined using a Multisizer 3 Coulter Counter (Beckman).

### Karyotype analysis

Exponentially growing ESCs were cultured in the presence of Karyo-MAX colcemid (Gibco) for 60 minutes, washed with PBS, trypsinized, and collected. ESCs were subsequently treated with hypotonic solution (75 mM KCl) for 6 minutes at 37°C, fixed with fixation solution (3 volumes Methanol, 1 volume Acetic acid), concentrated and dropped onto an angled, humidified microscope slide. The slide was dried and chromosomes were stained with Hoechst dye for 1 h in the dark. Images of metaphase spread were collected at a 60× objective on an Olympus Fluorescence Microscope.

### Rotary suspension culture and embryoid body differentiation

Embryoid bodies were formed by inoculating a single-suspension of ESCs that have been passaged without feeder layers for two generations (referred to as “day 0” culture) at 2×10^5^ cells/ml into 100 mm bacteriological grade polystyrene Petri dishes with 10 ml of differentiation media (DMEM, 15% FBS, 100 U/ml penicillin, 100 µg/ml streptomycin and 0.25 µg/ml amphotericin, 2 mM L-glutamine, 1× MEM non-essential amino acids, 0.1 mM β-mercaptoethanol). The EB cultures were immediately placed on rotary orbital shakers (Lab-Line Lab Rotator, Barnstead International) in a humidified incubator (37°C, 5% CO_2_) and maintained at 40–45 rpm for the entire duration of suspension culture; rotary speed was calibrated daily to ensure accuracy throughout. Rotary orbital culture has been shown previously to significantly enhance the efficiency, yield and homogeneity of EB populations compared to static suspension culture methods [18]. Differentiation media was exchanged every two days by collecting EBs via gravity-induced sedimentation in 15 ml conical tubes before aspirating spent media, replenishing with fresh media and returning the cultures to the rotary orbital shakers.

### RNA extraction and quantitative RT–PCR

Total RNA from ESCs and embryos was extracted with Trizol reagent (Invitrogen) and Allprep DNA/RNA Mini kit (Qiagen) respectively according to the manufacturer's instructions. RNA was reverse transcribed using a SuperScript III First-strand cDNA synthesis kit (Life Technologies). Real-time quantitative PCR (qPCR) were performed using iQ SYBR Green Supermix with MyIQ Single Color real-time PCR Detection System (Bio-Rad). The following primers were used: *Oct4*: forward 5′-GCTCA CCCTGGGCGTTCTC-3′, reverse 5′-GGCCGCAGCTTACACATGTTC-3′; *Nanog*: forward 5′-CCTCCAGCAGATGCAAGAACTC-3′, reverse 5′-CTTCAACCACTGGT TTTTCTGCC-3′; *Nkx2.5*: forward 5′-CAAGTGCTCTCCTGCTTTCC-3′, reverse 5′-GGCTTTGTCCAGCTCCACT-3′; alpha-MHC: forward 5′-GGTCCACATTCTTCA GGATTCTC-3′, reverse 5′-GCGTTCCTTCTCTGACTTTCG-3′; *Tyrosine hydroxylase*: forward 5′-GATTGCAGAGATTGCCTTCC-3′, reverse 5′-GGGTAGCATAGAGG CCCTTC-3′; *Nestin*: forward 5′-GCCTATAGTTCAACGCCCCC-3′, reverse 5′-AGAC AGGCAGGGCTAGCAAG-3′; *AFP*: forward 5′-AAACTCGCTGGAGTGTCTGC-3′, reverse 5′-AGGTTTGACGCCATTCTCTG-3′; *GFAP*: forward 5′-GCCACCAGT AACATGCAAGA-3′, reverse 5′-GGCGATAGTCGTTAGCTTCG; GAPDH: forward 5′-TTCACCACCATGGAGAAGGC-3′, reverse 5′-GGCATGGACTGTGGTCATGA-3′.

### PCR SuperArray analysis

RNA was isolated from ESC and EB samples using QIAshredders (as needed) and RNeasy Mini kits (Qiagen) according to the manufacturer's instructions. RNA quantity and quality were assessed by taking absorbance measurements at 260 and 280 nm on a NanoDrop ND1000 Spectrophotometer (Nanodrop Technologies). First strand cDNA synthesis was performed using the RT^2^ First Strand Kit (SABiosciences) with 1 µg of input RNA per well followed by real-time PCR using the Mouse Embryonic Stem Cells PCR SuperArray and SYBR Green RT^2^ qPCR Master Mix (SABiosciences), per manufacturer's recommended protocols. First strand synthesis and real-time PCR were performed using a BioRad MyCycler and BioRad MyIQ real time thermal cycler, respectively. Array results were first internally normalized to *GAPDH* levels and subsequently analyzed with Genesis software (Graz University of Technology) using log_2_ transformation, mean center gene analysis, and hierarchical clustering.

### Neural differentiation of ESCs

ESCs cultures were trypsinized with 0.25% trypsin-EDTA solution, depleted with feeder cells, and resuspended in differentiation media at 5×10^4^ cell/ml. Embryoid bodies were formed using hanging drop method by plating 20 µl drops (1000 cells per drop) on the inner side of the lid of 15 cm dishes. The bottom of the 15 cm dishes were filled with sterile water and incubated for 4 days. The neural differentiation protocol for ES cells was adapted from ES-Cult Neural differentiation protocols (StemCell Technologies, Vancouver, Canada). Briefly, four days old EBs were collected from the hanging drops and cultured for additional 2 days in 10 cm petri dishes in the presence of 1 µM *all-trans* retinoic acid. EBs were subsequently plated at 10 EBs per cm^2^ in tissue culture plates, coated with poly-L-ornithin and laminin (5 µg/ml), in NeuroCult NSC proliferation medium (StemCell Technologies) supplemented with FGF-b 10 ng/ml. The plates were incubated and the media was change every 2–3 days.

### Immunocytochemistry

Cells grown on glass cover slips were fixed with 4% paraformaldehyde for 20 min at room temperature before immunofluorescence staining. For immunocytochemistry, we used the following primary antibodies: GFAP (Abcam; rabbit IgG; 1∶1000), TUBB3 (Millipore; mouse IgG1; 1∶50); and secondary antibodies from Molecular Probes or Jackson Immuno Research Laboratories: Cy3-coupled donkey anti-rabbit, Alexa Fluor 488-coupled donkey anti-mouse antibodies. Nuclei were counter stained with Hoechst (1∶1000). Images were collected at 20× and 60× on an Olympus Fluorescence Microscope.

### Preparation and analysis of nuclei and histones of ESCs and EBs

mESC and EB nuclei and histones were prepared according to protocols described previously [10], [22], [23]. Briefly, cultured ESCs or EBs were harvested and nuclei were extracted using 0.5% Nonidet P-40 in RSB (10 mM NaCl, 3 mM MgCl_2_, 10 mM Tris-HCl, pH 7.5, protease inhibitors) and a Dounce homogenizer at 4°C. Released nuclei were pelleted and resuspended in RSB. Chromatin and histone proteins were subsequently extracted as described previously [22], [23]. 50–100 µg of total histone preparations were injected into a C18 reverse phase column (Vydac) on an ÄKTA UPC10 system (GE Healthcare). The effluent from the column was monitored at 214 nm (A_214_), and the peaks areas were recorded and determined with ÄKTA UNICORN 5.11 software. Relative amounts of total H1s were determined by ratio of the total A_214_ of all H1 peaks to half of the A_214_ of H2B peak. The A_214_ values of the H1 and H2B peaks were adjusted to account for the differences in the number of peptide bonds in each H1 subtype and H2B. Fractions corresponding to the H1d/H1e peak from HPLC analysis were collected and subjected to mass spectrometry analysis on a Qstar XL MS/MS system (Applied Biosystems) with electrospray ionization (ESI) as the ionization method. Analyst QS software (Applied Biosystems) was used for data acquirement and analysis.

### Mouse embryo preparation

H1c^+/−^H1d^+/−^H1e^+/−^ mice were set up for breeding in the afternoon, and embryos were staged as embryonic day 0.5 (E0.5) postcoitus at noon if a vaginal plug was found in the female in the next morning. The female was euthanized and embryos at E8.5 were dissected from the euthanized females according to procedures approved by Institutional Animal Care and Use Committee. DNA and RNA were extracted from embryos using Allprep DNA/RNA Micro kit (Qiagen) according to the manufacturer's instructions. Genotypes of embryos were determined by PCR assays described previously [15], [53].

### Quantitative chromatin immunoprecipitation (qChIP)

ChIP assays were performed as described previously [10] with modifications. Briefly, crosslinked chromatin was sheared to an average DNA fragment size of 200 to 400 bp by sonication. 20 µl of Dynabeads Protein G (Invitrogen) was incubated with 2 µg of antibody for 7 hours in 4°C. After washing three times with 1 ml PBS containing 0.5% BSA, the Dynabeads were then reacted with 40 µg of soluble chromatin overnight in 4°C. Dynabeads were washed five times with Washing Buffer (50 mM HEPES pH 7.6, 1 mM EDTA pH 8.0, 500 mM LiCl, 0.7% Sodium Deoxycholate, 1% NP-40) and one time with PBS. Protein/DNA complexes were subsequently eluted in 100 µl Elution Buffer (50 mM Tris-Cl pH 8.0, 10 mM EDTA pH 8.0, 1% SDS) at 65°C for 15 minutes, and incubated overnight at 65°C. DNA was purified with a Qiagen DNA Isolation column (Qiagen). The amount of each specific DNA fragment in immunoprecipitates was determined by real-time PCR. Triplicate PCR reactions using the iQ SYBR Green Supermix (BioRad) were analyzed in a MyIQ Real-Time PCR Detection System (BioRad). All samples were typically analyzed in triplicate in two independent experiments. The following primers were used: *Oct4*: forward 5′-TGGGCTGAAATACTGGGTTC-3′, reverse 5′- TTGAATGTTCGTGTGCCAAT-3′; *Nanog*: forward 5′-GGCATGGTGGTAGACAAGCC-3′, reverse 5′-TTAGTAAGTTGGTCCATGCTTTGG-3′. The percentage of input was calculated by dividing the amount of each specific DNA fragment in the immunoprecipitates by the amount of DNA present in the sample before immunoprecipitation (input DNA). The values from ChIP with control antibody (IgG) were typically less than 5% of the ChIP values with the antibodies against histone modifications.

### Antibodies

The following antibodies were used for Western blotting and qChIP: anti-OCT4 (Santa Cruz sc8628), anti-GAPDH (Ambion AM4300), anti-β ACTIN (Sigma-Aldrich A5316), anti-FLAG (Sigma-Aldrich F3165), anti-H1 (Millipore 05-457), anti-H1^0^ (Santa Cruz 56695), anti-H3K4me3 (Millipore 07-473), anti-H3K9me3 (Abcam 8898), anti-H3K27me3 (Millipore 07-449), anti-H3 (Abcam 1791) and IgG (Millipore 12-370).

### Bisulfite modification, PCR amplification, and sequencing analysis

Genomic DNA was prepared from mESCs, EBs, and embryos. 0.1 to 1 µg of DNA was treated with the Bisulfite Conversion Kit (CpG Genome) according to the manufacturer's manual. 1 µl of treated DNA was used in each PCR reaction as previously described [10]. The primers used to generate PCR products from the bisulfite-converted DNA are specific for the converted DNA sequence of the analyzed regions. The primer sequences were as follows: *Oct4* region1: forward 5′- GATATGGGTTGAAATATTGGGTTTAT-3′, reverse 5′-AATCCTCTCACCCCTACCTTAAAT-3′; *Oct4* region 2: forward 5′-AAGGTTGAAAATGAAGGTTTTTTG-3′, reverse 5′-TCCAACCATAAAAAAAATAAACACC-3′; *Nanog*: forward 5′- TTTGTAGGTGGGATTAATTGTGAAT-3′, reverse 5′-AAAAAATTTTAAACAACAACCAAAAA-3′. The PCR products were subsequently cloned using the TOPO TA Cloning kit (Invitrogen), and clones containing the converted DNA inserts were picked and sequenced. DNA sequences were analyzed with BiQ analyzer [54].

### Generation of H1d rescue (RES) cell lines

The H1d overexpression plasmid was constructed by cloning a 5 Kb fragment encompassing H1d coding region (with an insertion of FLAG tag at N-terminus) and proximal regulatory sequences into a vector containing a Blasticidin resistant gene. 20 µg of plasmid DNA was transfected into 2×10^7^ H1 TKO ESCs as described before [14], and 96 cell clones resistant to blasticidin were picked and analyzed by Western blotting using an anti-FLAG antibody (Sigma-Aldrich). Two cell lines with the highest levels of H1d were selected as RES cell lines for further analysis.

## Supporting Information

Figure S1 Chromosome spreads of WT and H1 TKO ESCs.
(TIF)

Figure S2 Gene expression analysis of ESCs and EBs formed in rotary suspension culture. (A) qRT-PCR analysis of expression levels of *Nkx2.5* and *α-MHC* in WT and H1 TKO cells during EB differentiation. Expression levels were normalized over *GAPDH*. (B) List of genes that displayed more than two-fold differences (P<0.05) in expression shown in Figure 2Di, 2Dii and 2Diii, respectively. (C) Scatter plot analysis comparing the degree of changes in gene expression in WT and H1 TKO cells during EB differentiation. X-axes and y- axes are delta delta CTs.
(TIF)

Figure S3 Analysis of total H1 and H1^0^ levels during EB differentiation. 2 µg histone proteins were analyzed with immunoblotting with antibodies indicated. The bottom panel of Western blotting with anti-H3 antibody demonstrates equal loading of proteins in each lane.
(TIF)

Figure S4 Increased expression of *Nanog* by H1 depletion in embryos. (A) qRT-PCR analysis of E8.5 embryos indicating the higher levels of *Nanog* expression in H1 TKO embryos compared with WT. Values are means ± SEM, n = 5 for each genotype. Expression levels were normalized over *GAPDH*. *: P<0.05. (B) DNA methylation status of promoter regions of *Nanog* in E8.5 embryos analyzed by bisulfite sequencing. (C) Percentage of CpG methylation calculated from results in (B).
(TIF)

Figure S5 Generation and characterization of RES ESC lines. (A) Representative Western blotting analysis of “rescue” clones. Immunoblotting with anti-β-ACTIN antibody indicates equal loading of whole cell lysates. (B) Reverse phase HPLC analysis of a RES cell line with high levels of H1d expression. (C) Chromosome spread of the RES cell shown in B). (D) Hematoxylin and eosin staining of sections of day 10 EBs generated from RES cells in rotary suspension culture. Scale bar: 100 µm. (E) qRT-PCR analysis of differentiation markers in RES cells during EB differentiation. Expression levels were normalized over *GAPDH*.
(TIF)

Figure S6 Analysis of expression and epigenetic marks at *Nanog* promoter. (A) qRT-PCR analysis of *Nanog* expression in ESCs and day 10 EBs. Expression levels were normalized over *GAPDH*. (B) DNA methylation status of *Nanog* promoter in mouse embryonic fibroblasts (MEFs) (left) or in ESCs (day 0) and day 10 EBs (right). (C) qChIP analysis of H1, H3K4me3, H3K9me3 and H3K27me3 levels at *Nanog* promoters in ESCs (day 0) and day 10 EBs. Data were normalized as described in Figure 5Biv. *: P<0.05; **: P<0.01.
(TIF)

Figure S7 qChIP Analysis of H1^0^ occupancy at *Oct4* promoter during EB differentiation. Data were normalized as described in Figure 5Biv. *: P<0.05; **: P<0.01; ***: P<0.001.
(TIF)
