# Supplementary figures and images for: Histone H1 Depletion Impairs Embryonic Stem Cell Differentiation
Source: PLoS Genet. 2012 May 10;8(5):e1002691. doi: 10.1371/journal.pgen.1002691 (PMC3349736; doi:10.1371/journal.pgen.1002691)

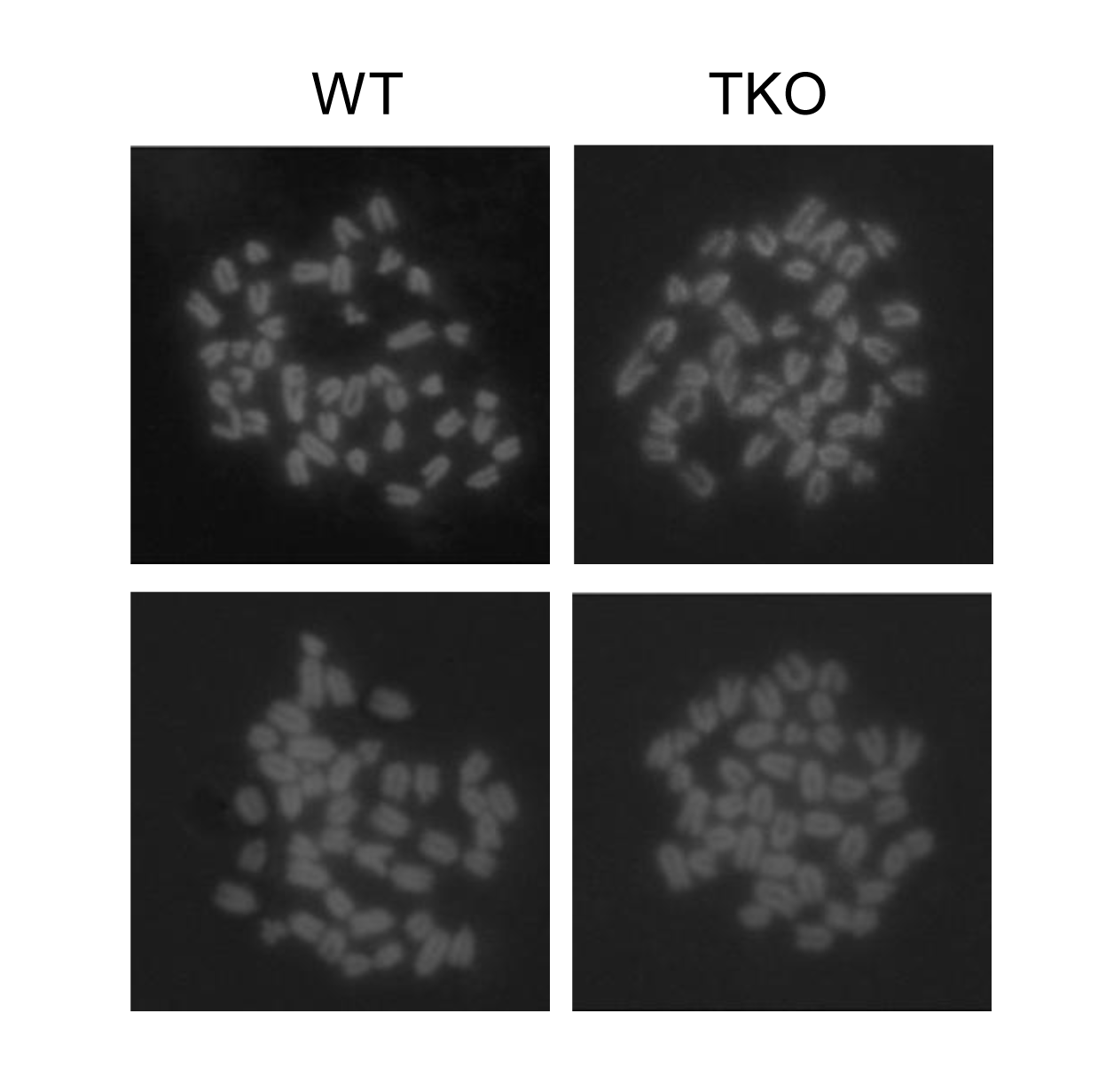

Supplement: Figure S1 — Chromosome spreads of WT and H1 TKO ESCs. (TIF) [file pgen.1002691.s001.tif]

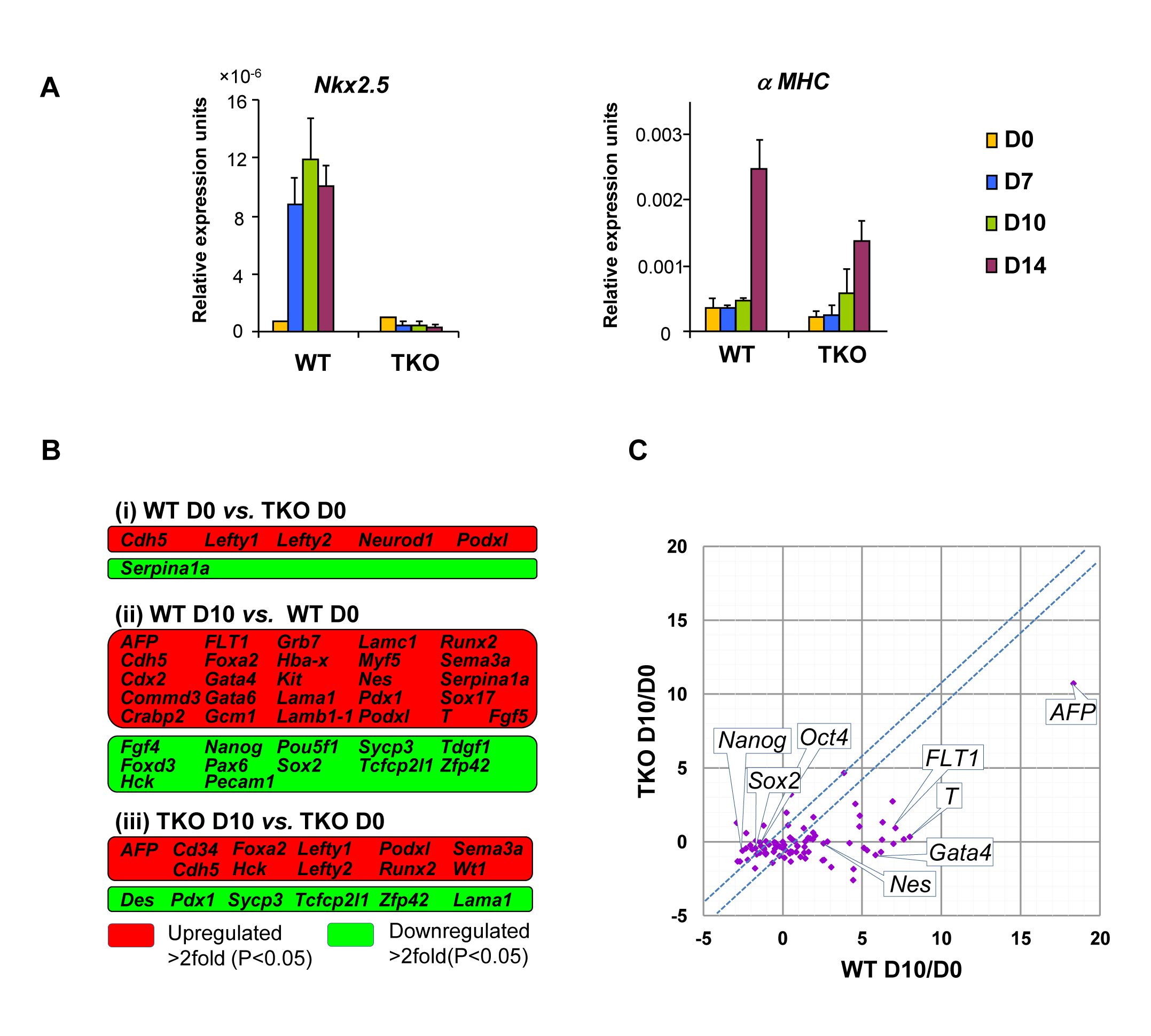

Supplement: Figure S2 — Gene expression analysis of ESCs and EBs formed in rotary suspension culture. (A) qRT-PCR analysis of expression levels of Nkx2.5 and α-MHC in WT and H1 TKO cells during EB differentiation. Expression levels were normalized over GAPDH. (B) List of genes that displayed more than two-fold differences (P<0.05) in expression shown in Figure 2Di, 2Dii and 2Diii, respectively. (C) Scatter plot analysis comparing the degree of changes in gene expression in WT and H1 TKO cells during EB differentiation. X-axes and y- axes are delta delta CTs. (TIF) [file pgen.1002691.s002.tif]

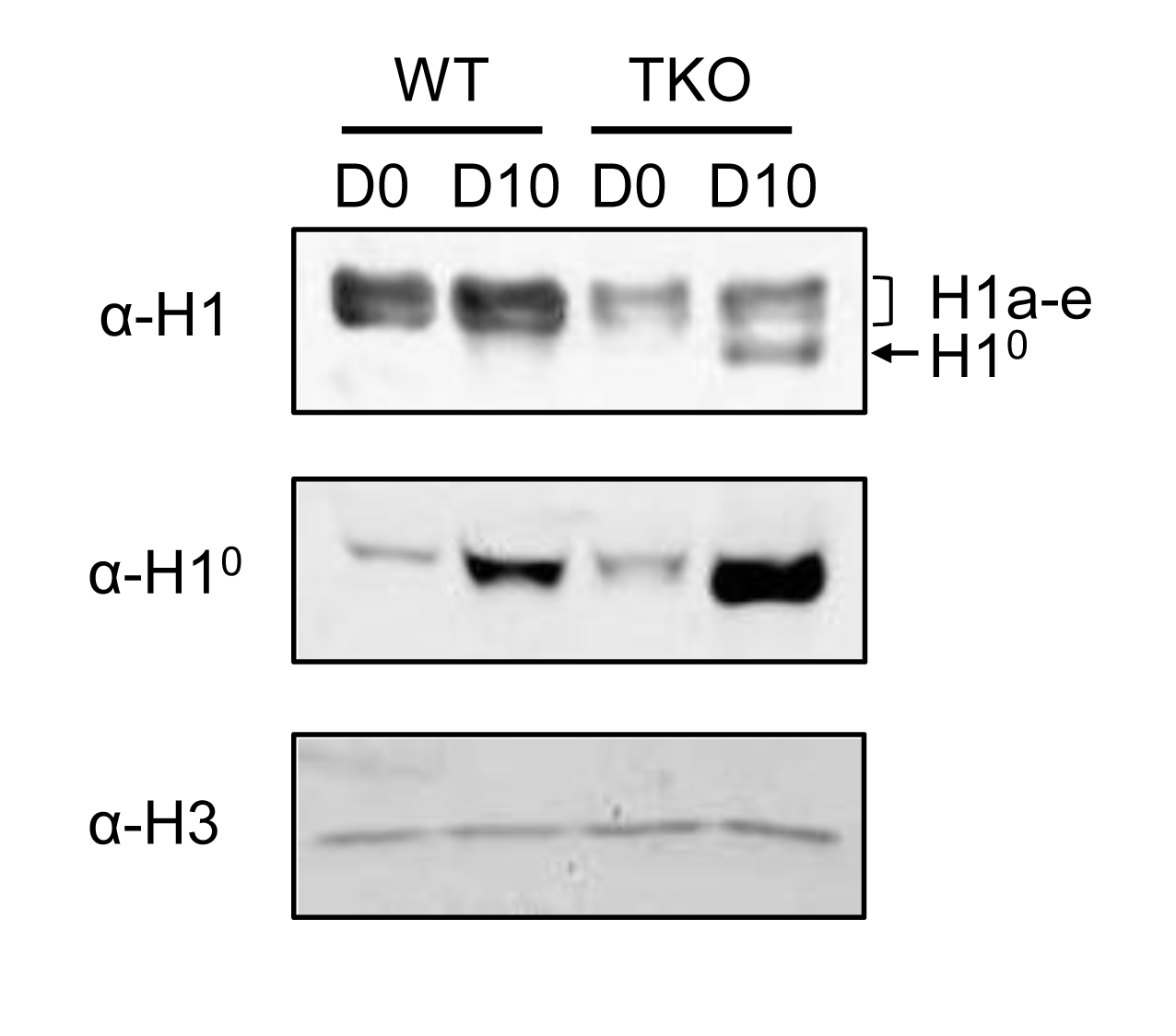

Supplement: Figure S3 — Analysis of total H1 and H10 levels during EB differentiation. 2 µg histone proteins were analyzed with immunoblotting with antibodies indicated. The bottom panel of Western blotting with anti-H3 antibody demonstrates equal loading of proteins in each lane. (TIF) [file pgen.1002691.s003.tif]

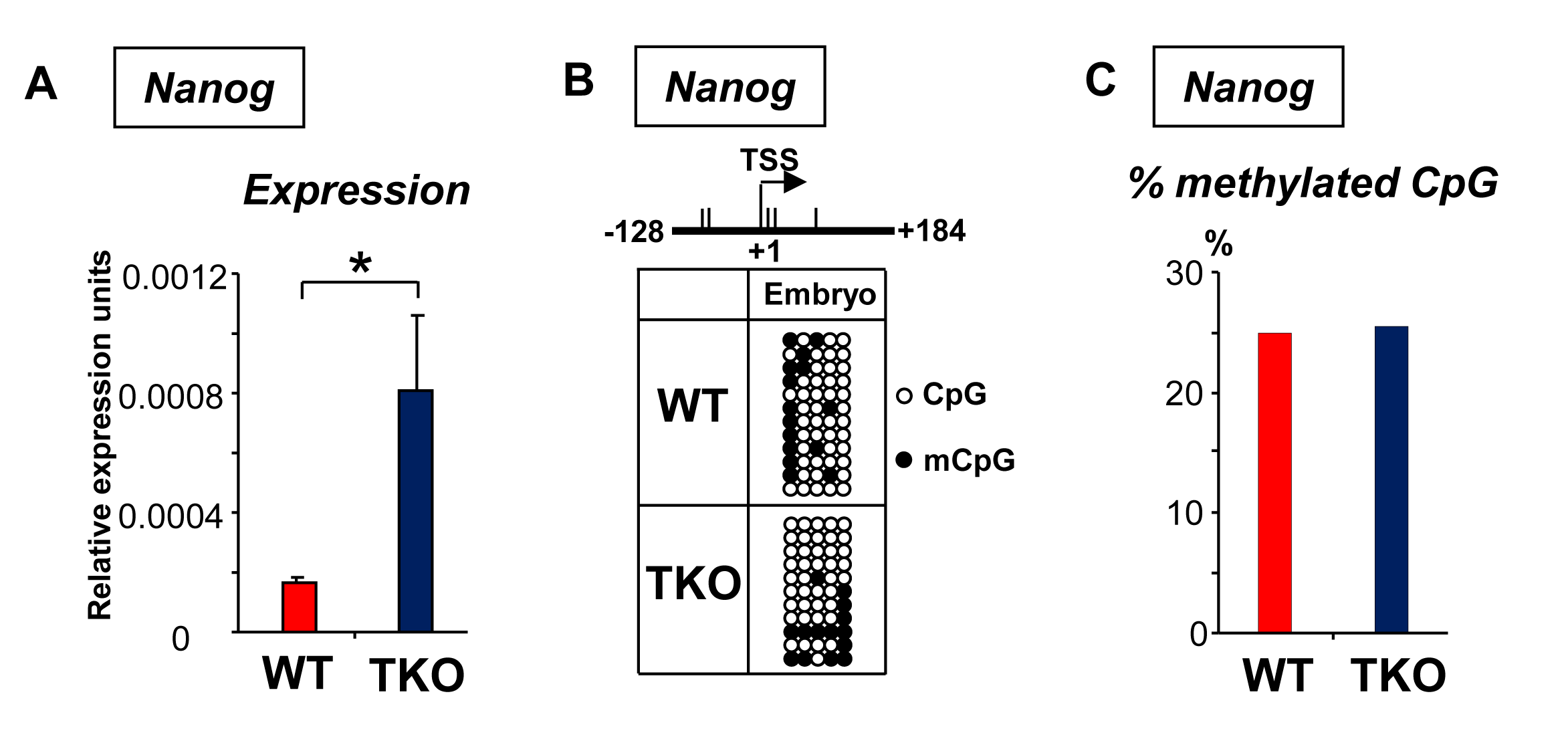

Supplement: Figure S4 — Increased expression of Nanog by H1 depletion in embryos. (A) qRT-PCR analysis of E8.5 embryos indicating the higher levels of Nanog expression in H1 TKO embryos compared with WT. Values are means ± SEM, n = 5 for each genotype. Expression levels were normalized over GAPDH. *: P<0.05. (B) DNA methylation status of promoter regions of Nanog in E8.5 embryos analyzed by bisulfite sequencing. (C) Percentage of CpG methylation calculated from results in (B). (TIF) [file pgen.1002691.s004.tif]

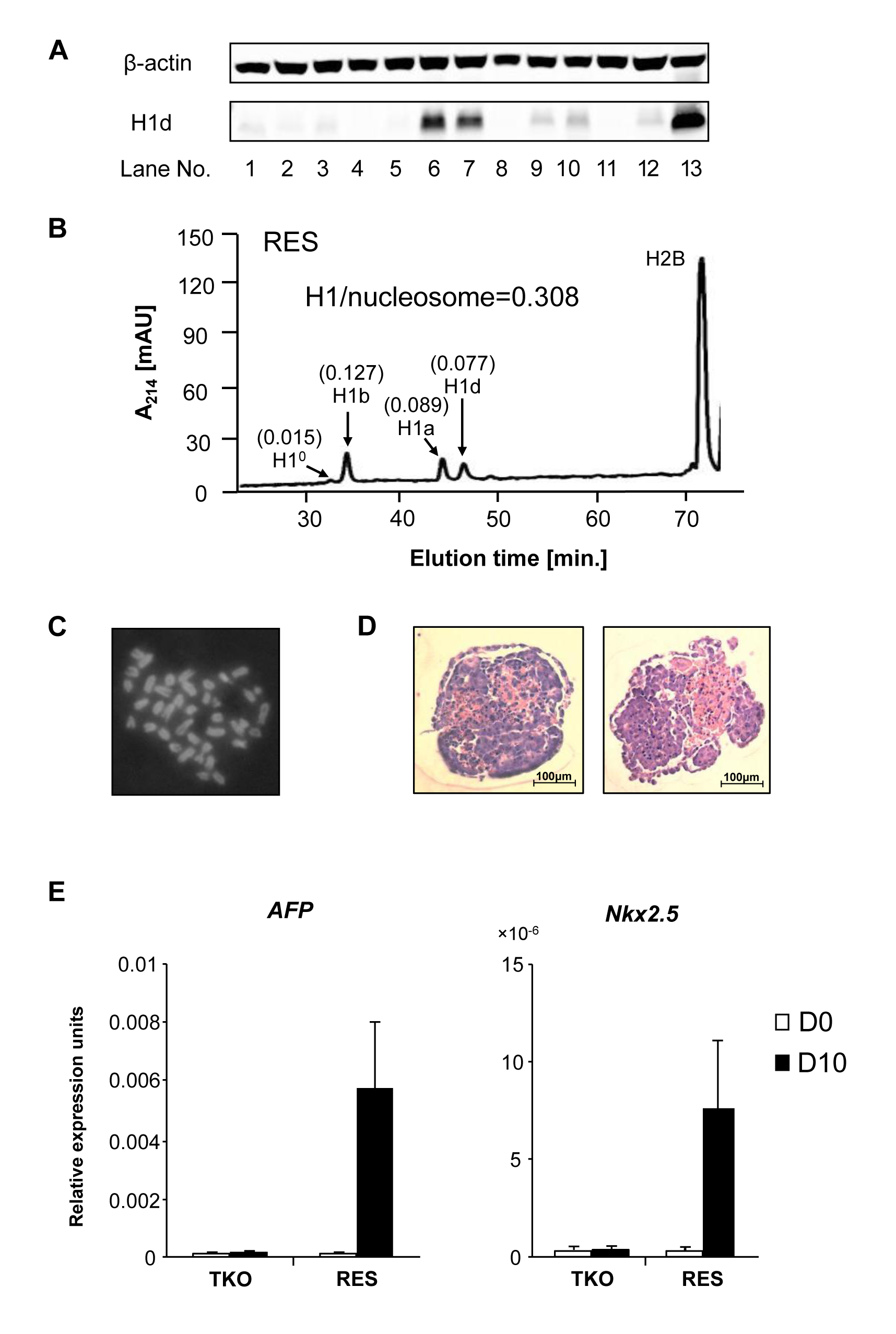

Supplement: Figure S5 — Generation and characterization of RES ESC lines. (A) Representative Western blotting analysis of “rescue” clones. Immunoblotting with anti-β-ACTIN antibody indicates equal loading of whole cell lysates. (B) Reverse phase HPLC analysis of a RES cell line with high levels of H1d expression. (C) Chromosome spread of the RES cell shown in B). (D) Hematoxylin and eosin staining of sections of day 10 EBs generated from RES cells in rotary suspension culture. Scale bar: 100 µm. (E) qRT-PCR analysis of differentiation markers in RES cells during EB differentiation. Expression levels were normalized over GAPDH. (TIF) [file pgen.1002691.s005.tif]

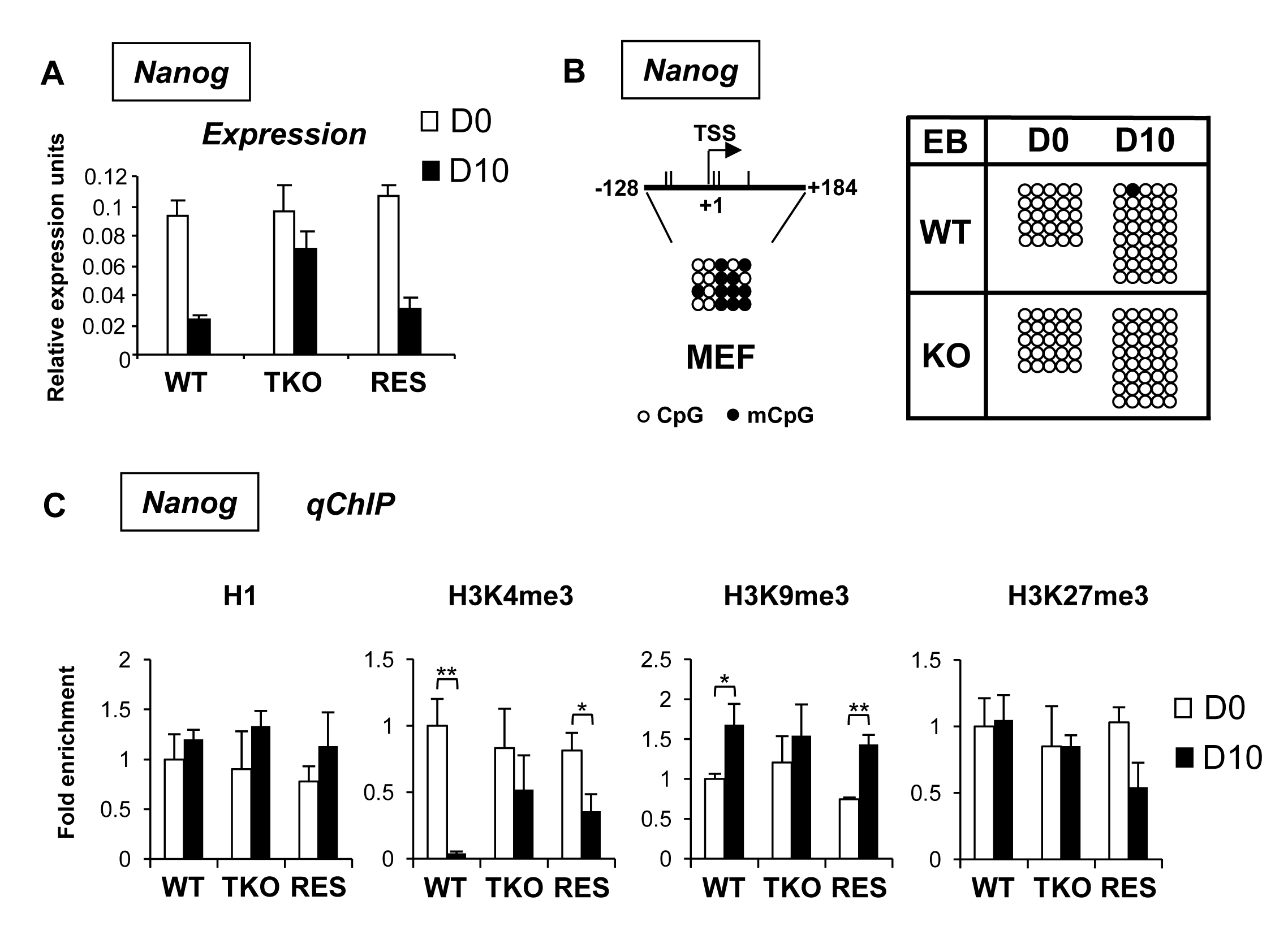

Supplement: Figure S6 — Analysis of expression and epigenetic marks at Nanog promoter. (A) qRT-PCR analysis of Nanog expression in ESCs and day 10 EBs. Expression levels were normalized over GAPDH. (B) DNA methylation status of Nanog promoter in mouse embryonic fibroblasts (MEFs) (left) or in ESCs (day 0) and day 10 EBs (right). (C) qChIP analysis of H1, H3K4me3, H3K9me3 and H3K27me3 levels at Nanog promoters in ESCs (day 0) and day 10 EBs. Data were normalized as described in Figure 5Biv. *: P<0.05; **: P<0.01. (TIF) [file pgen.1002691.s006.tif]

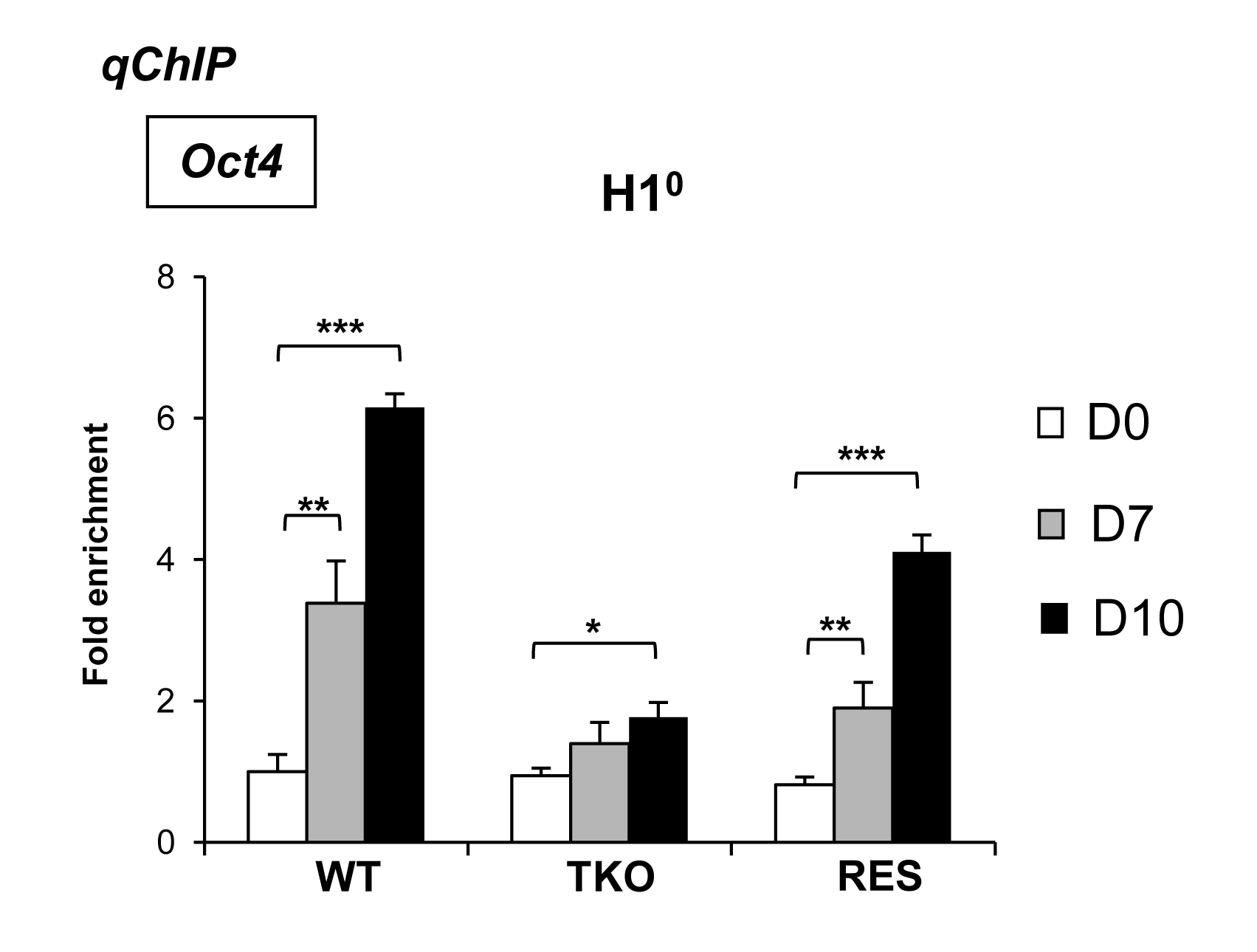

Supplement: Figure S7 — qChIP Analysis of H10 occupancy at Oct4 promoter during EB differentiation. Data were normalized as described in Figure 5Biv. *: P<0.05; **: P<0.01; ***: P<0.001. (TIF) [file pgen.1002691.s007.tif]
